# Constructing a One Health governance architecture: a systematic review and analysis of governance mechanisms for One Health

**DOI:** 10.1093/eurpub/ckae124

**Published:** 2024-08-30

**Authors:** Darlington David Faijue, Allison Osorio Segui, Kalpita Shringarpure, Ahmed Razavi, Nadeem Hasan, Osman Dar, Logan Manikam

**Affiliations:** Aceso Global Health Consultants Pte Ltd, Singapore, Singapore; Institute for Infection and Immunity Research, St George’s, University of London, London, United Kingdom; Purdue University Global, Health Sciences and Military Physician Assistant Preparation (MPAP), Indiana, United States; Aceso Global Health Consultants Pte Ltd, Singapore, Singapore; Department of Community Medicine, Medical College Baroda, Vadodara, India; IHR Strengthening Project, Global Operations, UK Health Security Agency, London, United Kingdom; Foreign, Commonwealth and Development Office, London, United Kingdom; Chatham House Centre for Universal Health, London, United Kingdom; Aceso Global Health Consultants Pte Ltd, Singapore, Singapore; Chatham House Centre for Universal Health, London, United Kingdom; Department of Epidemiology and Public Health, Institute of Epidemiology and Health Care, University College London (UCL), London, United Kingdom

## Abstract

The integration of human, animal, and environmental health in the One Health framework is crucial for tackling complex health and environmental issues. Governance structures in One Health initiatives are essential for coordinating efforts, fostering partnerships, and establishing effective policy frameworks. This systematic review, registered with PROSPERO, aims to evaluate governance architectures in One Health initiatives. Searches in PubMed, Scopus, WoS, and Cochrane from 2000 to 2023 were conducted. Key terms focused on peer-reviewed articles, systematic reviews, and relevant grey literature. Nine eligible studies were selected based on inclusion criteria. Data synthesis aimed to assess governance mechanisms’ functionality and effectiveness. Among 1277 sources screened, nine studies across diverse regions were eligible. An adapted framework assessed implementation mechanisms of international agreements, categorizing them into Engagement, Coordination, Policies, and Financial domains. The findings highlight the importance of effective governance, stakeholder engagement, and collaborative approaches in addressing One Health’s challenges. Identified challenges include deficient intersectoral collaboration, funding constraints, and stakeholder conflicts. Robust governance frameworks are pivotal in One Health paradigms, emphasizing stakeholder engagement and collaboration. These insights guide policymakers, practitioners, and researchers in refining governance structures to enhance human-animal health and environmental sustainability. Acknowledging study limitations, such as methodological variations and limited geographical scope, underscores the importance of further research in this area.

## Introduction

In recent years, the One Health concept has gained considerable attention, especially with the launch of initiatives like the One Health Joint Plan of Action (2022–2026) [1]. Spearheaded by Quadripartite Organizations such as the FAO, UNEP, WOAH, and WHO, this collaborative effort addresses health challenges at the intersection of human, animal, and environmental health on a global scale [1]. The One Health approach recognizes that the health of humans, animals, and the environment are intricately interconnected and that addressing health challenges requires a coordinated, multidisciplinary approach. The One Health Joint Plan of Action serves as a strategic framework for promoting collaboration among various stakeholders and enhancing capacities to manage complex health risks, strengthen health systems, and ensure environmental sustainability [1, 2].

### Evolution and principles of One Health

The concept of One Health emerged from the recognition of the interconnectedness of human, animal, and environmental health [3]. It seeks to address health challenges holistically by considering the interdependencies between these three domains. The operational definition provided by the One Health High-Level Expert Panel-OHHLEP aims to facilitate a clear understanding across various sectors and areas of expertise [3]. This definition serves as a foundational set of guiding principles adaptable to specific stakeholders, assisting in outlining the general considerations for a One Health approach and fostering innovation, cooperation, and collaboration across relevant sectors and disciplines. While topics like food and water security, energy, and environmental/ecosystem health possess sector-specific and specialized concerns extending beyond the scope of One Health, their intersection represents shared responsibilities crucial for protecting and addressing health challenges.

### Interconnectedness of health domains

The fundamental premise of One Health lies in acknowledging the intricate interconnections between human, animal, and environmental health [3]. Human health is deeply influenced by animal health and the health of ecosystems [4]. Zoonotic diseases, for instance, often originate in animals and can spill over to humans, posing significant public health threats [2, 5]. These diseases, such as Ebola and avian influenza, exemplify the importance of a unified approach to health that encompasses multiple sectors [2, 5]. The interconnectedness of health domains underscores the need for collaborative efforts to address health challenges comprehensively.

### Role of governance mechanisms in One Health

Effective governance mechanisms are essential for facilitating collaboration and coordination among stakeholders involved in One Health initiatives [3, 5–7]. Governance within the context of One Health is defined as the establishment of structured mechanisms and processes that enable effective coordination, collaboration, and decision-making among diverse stakeholders. These mechanisms encompass policies, regulations, and frameworks that guide the integration of multiple disciplines and sectors, including medicine, veterinary science, ecology, epidemiology, and environmental science. Key components of governance encompass the establishment of clear roles and responsibilities, mechanisms for communication and information sharing, as well as the promotion of transparency and accountability.

### Studies on One Health governance mechanisms

Numerous studies have delved into the governance architectures deployed within One Health initiatives, emphasizing stakeholder engagement [8, 9], policy frameworks [10, 11], and collaborative strategies [12, 13]. These studies provide insights into the various governance mechanisms employed, as well as the challenges encountered in implementing them. For instance, Huang *et al*. [12] explored health system strengthening and hypertension management in China, emphasizing coordinated governance structures for sustainable health outcomes. Bordier *et al*. [13] conducted a systematic review characterizing One Health surveillance systems, highlighting cross-disciplinary approaches and collaborative strategies as pivotal components. Simen-Kapeu *et al*. [8] focused on strengthening community health programs in Liberia, stressing the importance of stakeholder engagement within governance structures.

### Challenges and opportunities

Despite its potential, implementing One Health approaches poses challenges such as inadequate funding, jurisdictional conflicts, and the need for enhanced cross-disciplinary communication and education [14, 15]. However, the approach offers significant opportunities for addressing emerging infectious diseases, promoting food security, conserving biodiversity, and mitigating environmental degradation [1, 5, 16, 17].

### Global relevance and adoption of One Health

The global relevance of One Health is increasingly recognized, leading to its incorporation into national and international policies and strategies [2, 18–21]. Many nations globally have acknowledged the significance of One Health and have instituted specialized One Health Networks (OHNs) [22]. These networks play a critical role in averting misalignments in investments designated for One Health and minimizing duplications while addressing health challenges holistically [2, 18–22]. Additionally, the Sustainable Development Goals (SDGs), particularly SDG 3 (Good Health and Well-being), SDG 6 (Clean Water and Sanitation), SDG 13 (Climate Action), and SDG 15 (Life on Land), align with the principles of One Health, emphasizing the interconnectedness of health and ecosystems [8, 23].

## Methods

This Systematic Review, registered with PROSPERO (ID: CRD42018086843) on 26 January 2018 and updated on 18 December 2020, adheres to PRISMA guidelines for systematic reviews [24, 25]. We conducted a comprehensive search across PubMed, Scopus, and Web of Science, focusing on peer-reviewed articles, reports, and grey literature about governance structures in One Health practices.

### Search strategy

The search strategy combined controlled vocabulary terms and keywords (MeSH terms) related to “One Health governance” to retrieve relevant articles. Boolean operators “AND” and “OR” ensured comprehensive results. The search terms included: (“One Health governance” OR “Interdisciplinary health governance” OR “Governance mechanisms for One Health” OR “Integrated health governance” OR “Policy frameworks for One Health” OR “Collaborative strategies in One Health” OR “Stakeholder engagement in One Health” OR “Environmental health governance” OR “Human-animal health governance” OR “Public health policy and One Health” OR “Global health governance strategies”). Detailed MeSH Terms and Keywords searches are provided in Supplementary Table S1.

### Inclusion and exclusion criteria

Criteria were established to guide the selection process, including publication type, publication date, language, content relevance, and study type. Predetermined inclusion and exclusion criteria are listed below:

#### Inclusion criteria

- **Publication type:** Peer-reviewed articles, systematic reviews, meta-analyses, reports, and relevant grey literature on governance structures, policies, frameworks, or strategies within One Health practices.
- **Publication date:** Studies published from January 2000 to December 2023 to cover contemporary literature and the evolution of One Health initiatives.
- **Language:** English to ensure comprehensive comprehension and synthesis.
- **Content relevance:** Articles addressing governance mechanisms within One Health, focusing on stakeholder engagement, policy frameworks, institutional arrangements, collaborative strategies, or challenges.
- **Study type:** Qualitative, quantitative, or mixed-method studies on governance mechanisms, structures, or strategies integrating human, animal, and environmental health.

#### Exclusion criteria

- **Non-English publications:** To ensure comprehensive comprehension.
- **Non-relevant content:** Publications not directly addressing governance mechanisms within the One Health framework.
- **Irrelevant study types:** Studies not related to governance structures, policies, frameworks, or strategies in One Health.
- **Publication date:** Studies published before 2000 or after December 2023 to focus on contemporary research, as the One Health approach started to gain momentum during this time frame.
- **Publication type:** Conference abstracts, editorials, opinion pieces, and non-peer-reviewed publications lacking empirical data or analysis.

### Study selection

Two independent reviewers screened studies from relevant databases (e.g. PubMed, Scopus, Cochrane, and Web of Science) based on titles and abstracts, with full-text articles evaluated for eligibility. Discrepancies were resolved through discussion with a third reviewer. The search strategy aimed to encompass peer-reviewed articles, systematic reviews, meta-analyses, reports, and relevant grey literature discussing governance structures, policies, frameworks, or strategies associated with One Health practices. Inclusion criteria focused on peer-reviewed articles, systematic reviews, meta-analyses, reports, and relevant grey literature published in English between January 2000 and December 2023. The timeframe of 2000–2023 was selected because The One Health approach gained momentum from 2000 to 2023 due to key events such as the rise in zoonotic diseases (SARS, H5N1), and initiatives such as the One Health Initiative (2007) and Global Health Security Agenda (2014), highlighting its importance in integrated health efforts.

### Data extraction and quality assessment

Standardized forms were used to extract information from selected studies, summarized using Stata/IC 17.1. Quality assessment with the Joanna Briggs Institute tool ensured reliability (Table 1). Studies were categorized by ownership, geographic scope, recognition of One Health, and sector suitability by two authors (DDF, AOS). These were coded and analysed using an adapted framework for assessing international agreement implementation mechanisms, determining the best-fit category for each domain. Domains were supported by evidence and recommendations for effective One Health governance and sustainability.

- **Engagement mechanism:** Refers to legal instruments in governance, either as soft law (action plans, agreements, conventions, guidelines, recommendations, or strategies) or hard law (directives, legislation, or regulations). Elnaiem *et al*. [37] highlight the importance of enforceable legislation and recommendations, emphasizing soft law instruments like guidelines and strategies. Horefti [38] stresses the need for preparedness and mitigation strategies.
- **Coordination mechanism:** Methods or processes to facilitate governance within One Health, including peer review, expert review, self-reports, civil society involvement, and decision-making. Sinclair [39] proposes enhanced coordination and data sharing for effective governance. Mazet *et al*. [40] emphasize regional networks in improving disease surveillance and coordinated interventions, highlighting civil society involvement.
- **Policy mechanisms:** Administrative structures in the One Health framework, involving international organizations, joint ventures, or sub-agencies, critical in implementing and enforcing policies. Aslam *et al*. [41] discuss national action plans and international organizations’ roles in promoting policy, advocacy, and antimicrobial stewardship. Keusch *et al*. [42] apply lessons from RNA virus outbreaks to pandemic preparedness, supporting international organizations.
- **Financial mechanism:** Approaches to manage funds and resources for One Health programs, such as membership organizations, coordinated self-management, multilateral funds, and donor grants. Elnaiem *et al*. [37] advocate for increased investment through multilateral funds and donor grants. Espeschit *et al*. [43] examine challenges in implementing One Health strategies in Brazil, suggesting improved financial mechanisms for monitoring across sectors.

## Results

### Synthesis and reporting

The synthesis of findings from the selected studies involved systematic categorization and analysis, within these distinct themes: engagement mechanism, coordination mechanism, policies mechanism, and financial mechanism. These thematic areas underscored the pivotal roles of stakeholders [9], the efficacy of policies [8, 26], institutional arrangements [12, 13], collaborative strategies [12], and challenges encountered by governance structures within One Health initiatives [8, 9, 12, 13]. Employing a narrative synthesis approach, a comprehensive overview of the identified governance mechanisms was conducted, adhering to the standards for Synthesis and Reporting in systematic reviews.

**Table 1.** JBI quality assessment and characteristics of the included studies.

First author (Reference)	Publication year	Country	Methods	Main findings	Quality assessment
Huang *et al*. [12]	2016	China	Qualitative analysis, environmental considerations, health outcome assessments	Coordinated governance structures were pivotal, highlighting the necessity of collaborative frameworks for sustainable health outcomes, especially in managing chronic health conditions	Moderate to high quality
Dahal *et al*. [27]	2017	South Asia (40)	Literature reviews, stakeholder consultations	The study underscored the significance of cross-sectoral collaboration and stakeholder engagement within governance frameworks for effective implementation of One Health approaches	Moderate to high quality
Rajan *et al*. [9]	2019	Thailand	Participatory action research, stakeholder workshops	Highlighted the role of participatory governance structures in ensuring community engagement and inclusivity in health decision-making processes	Moderate to high quality
Bordier *et al*. [13]	2020	Globally	Systematic review, cross-disciplinary literature analysis	Emphasized effective cross-disciplinary approaches and collaborative strategies within governance frameworks for successful surveillance systems and emphasized the need for integrated approaches and stakeholder collaboration	High quality
Simen-Kapeu *et al*. [8]	2021	Liberia	Qualitative surveys, stakeholder consultations	Stressed stakeholder engagement within governance structures to enhance health system resilience, particularly in resource-constrained settings. Highlighted the importance of participatory approaches within governance frameworks.	Moderate to high quality
Li *et al*. [28]	2021	China	Field observations, case studies	Highlighted the imperative of integrated governance systems involving multiple sectors and stakeholders for effective zoonotic disease management	Moderate to high quality
Abbas *et al*. [10]	2022	Globally	Qualitative interviews, case studies	Emphasized collaborative governance structures and stakeholder engagement as fundamental components for successful One Health initiatives	Moderate to high quality
Aggarwal *et al*. [11]	2022	Globally	Mixed-methods surveys, interviews	Emphasized stakeholder engagement and collaboration within governance structures for patient-centered care and effective health system governance	Moderate to high quality
Liu *et al*. [33]	2023	BRICS Countries (41)	Policy analysis, expert consultations	Underscored the pivotal role of governance frameworks and policies in fostering health collaboration across nations, emphasizing collaborative governance mechanisms within the One Health paradigm	High quality

It summarizes the methodology, key findings, and quality assessment of each study within the systematic review context. The included studies exhibited moderate to high-quality assessments, reflecting their methodological rigor and comprehensive analysis. Huang *et al*. [12] conducted thorough qualitative analysis, emphasizing collaborative governance structures for sustainable health outcomes in China. Dahal *et al*. [27] highlighted cross-sectoral collaboration and stakeholder engagement in effective One Health approaches in South Asia. Rajan *et al*. [9] emphasized participatory governance structures for community engagement in health decision-making processes in Thailand. Bordier *et al*. [13] Emphasized effective cross-disciplinary approaches and collaborative strategies within governance frameworks for successful surveillance systems and emphasized the need for integrated approaches and stakeholder collaboration. Simen-Kapeu *et al*. [8] stressed stakeholder engagement for health system resilience in Liberia. Li *et al*. [28] underscored integrated governance systems for zoonotic disease management in China. Abbas *et al*. [10] emphasized collaborative governance structures for successful One Health initiatives globally. Aggarwal *et al*. [11] highlighted stakeholder engagement for patient-centered care and effective health system governance globally. Liu *et al*. [33] emphasized governance frameworks and policies for health collaboration across BRICS countries.

### Outcomes

**Figure 1.**
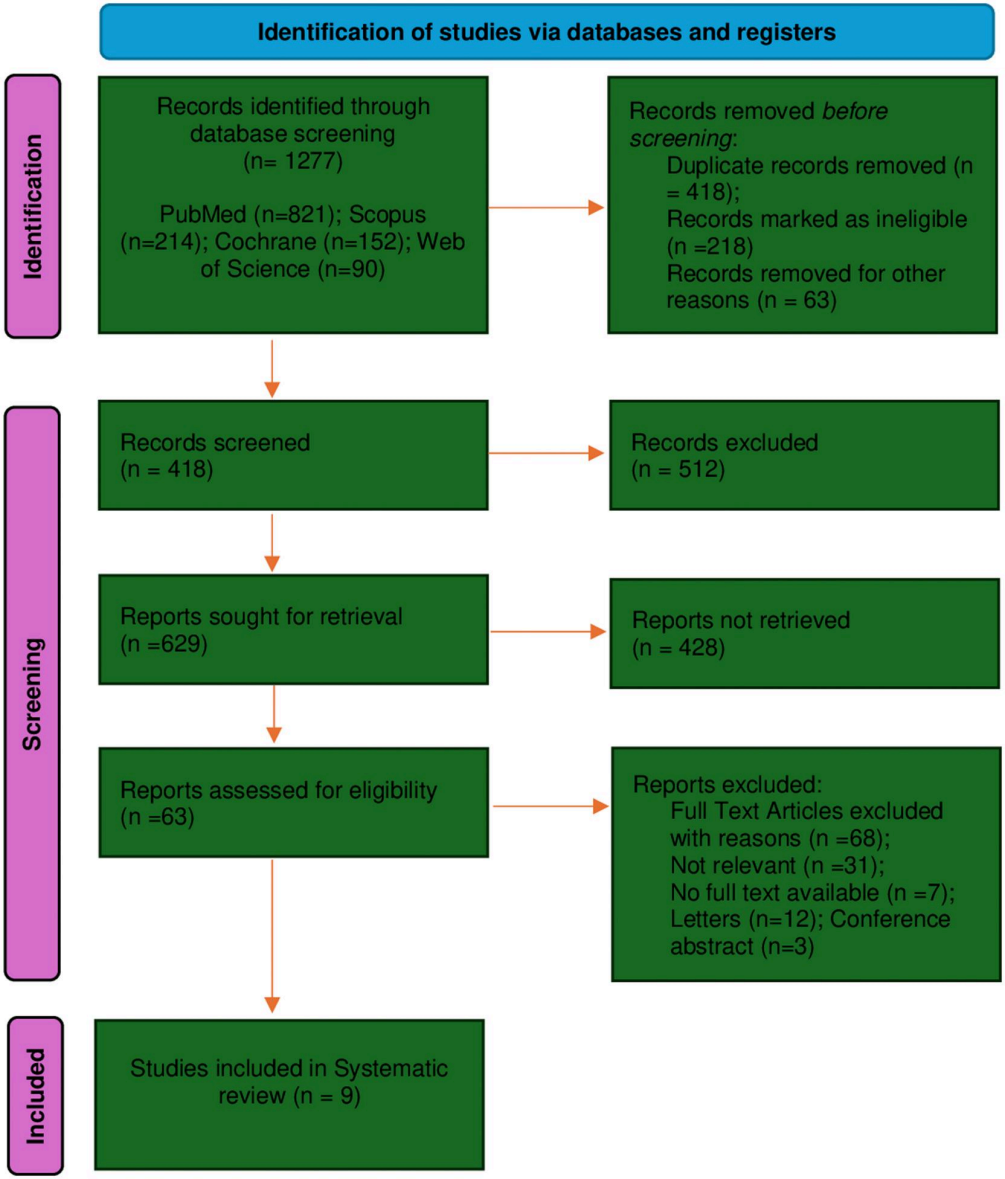
PRISMA chart.

After reviewing 1277 scholarly publications, only 9 articles met the criteria for this systematic review. Spanning from 2016 to 2023 (Figure 1), these studies covered various regions including China, Liberia, Thailand, BRICS nations, and South Asia [8, 9, 12, 13, 26]. Each article sheds light on One Health, particularly focusing on governance mechanisms and strategies. They emphasize the importance of effective governance structures, stakeholder engagement, and collaborative approaches in One Health initiatives. The studies involved diverse demographics and methodologies. For instance, Huang *et al*. conducted qualitative analyses on coordinated governance structures’ impact on chronic health conditions [8]. Dahal *et al*. emphasized cross-sectoral collaboration through literature reviews and stakeholder consultations [27]. Rajan *et al*. ensured community engagement in health decision-making via participatory action research [9].

The studies used diverse methodologies and demographics. Huang *et al*. [8] conducted qualitative analyses on the impact of coordinated governance structures on chronic health conditions in China, highlighting positive outcomes. Dahal *et al*. [27] emphasized the importance of cross-sectoral collaboration through literature reviews and stakeholder consultations in South Asia. Rajan *et al*. [9] demonstrated the benefits of community engagement in health decision-making within BRICS nations via participatory action research. Bordier *et al*. [13] highlighted collaborative strategies in governance frameworks for successful surveillance systems through a systematic review. Simen-Kapeu *et al*. [8] emphasized stakeholder engagement for enhanced health system resilience. Li *et al*. [28] underscored integrated governance for effective zoonotic disease management. Abbas *et al*. [10] and Aggarwal *et al*. [11] highlighted collaborative governance for One Health initiatives and patient-centred care, respectively. Liu *et al*. [14] emphasized governance frameworks in fostering health collaboration.

## Discussion

### Summary of key findings

The systematic review extensively investigates the multifaceted landscape of One Health governance frameworks, revealing the complex roles played by coordinated governance structures [28], active stakeholder engagement [9], methodically designed policy frameworks [8], and concerted collaborative strategies [12] across various studies. This analysis underscores the intertwined and interdependent nature of these components, emphasizing their collective interaction in facilitating successful health outcomes across diverse sectors. The reviewed studies also collectively highlight the critical importance of integrated approaches in addressing complex health issues. They advocate for holistic methodologies in public health, stressing the necessity for interdisciplinary strategies in both academia and policy formulation [28]. This collective emphasis resonates throughout the domains of public health research and policy, indicating a need for a paradigm shift towards comprehensive, interconnected approaches that transcend disciplinary boundaries.

Through synthesizing insights from the diverse studies included in this review, an evident narrative emerges, highlighting the indispensable nature of cohesive governance structures [13] as foundational elements for effective collaboration [8]. These findings underscore the pivotal role of engaged stakeholders [9] as catalysts in propelling progress within such frameworks. Supported by astutely crafted policy frameworks [8], these governance structures establish the groundwork for resilient and adaptive health systems capable of navigating modern health challenges intricately [13].

### One Health governance definition

Governance encompasses the processes, structures, and institutions managing a healthcare system or other systems [29]. It involves actors like government agencies, healthcare providers, patients, and civil society organizations, aiming for equitable, efficient, and high-quality services. One Health Governance integrates human, animal, plant, and environmental health principles at their intersection [1, 29].

Comprising strategic frameworks, oversight mechanisms, and stakeholder engagement, One Health Governance seeks to balance human, animal, and ecosystem health sustainably. Effective governance is crucial for managing chronic conditions [1, 8, 14], optimizing surveillance [17, 30], fortifying resilience [23, 30], and ensuring equity [31, 32].

#### Explanation of Fig. 2: Core of one health governance with key elements and challenges

**Figure 2.**
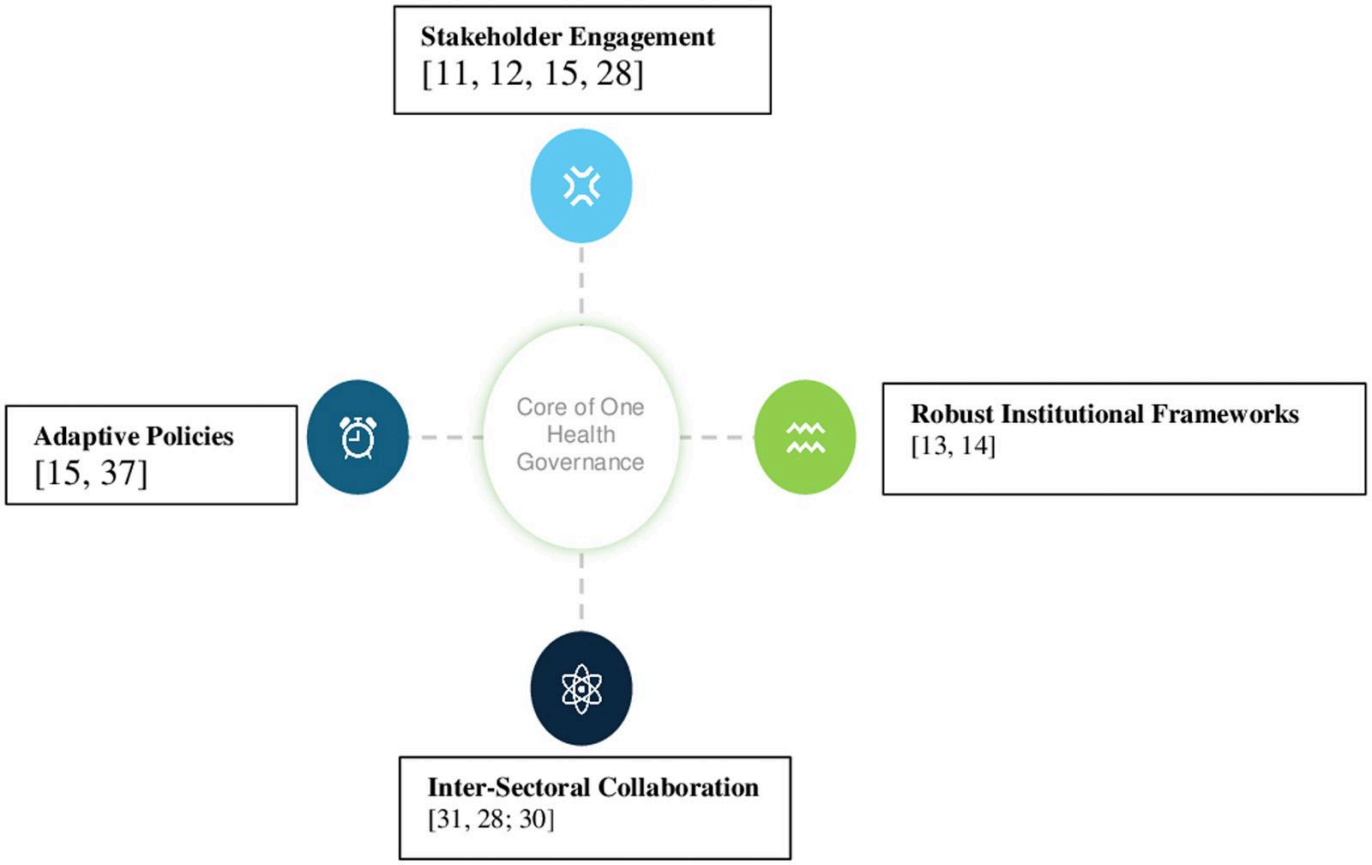
Core of one health governance with key elements and challenges.

In Fig. 2, the core of One Health Governance is delineated, encompassing four major areas: Stakeholder Engagement [8, 10, 11, 13], Robust Institutional Frameworks [12, 13], Adaptive Policies [8, 33], and Inter-Sectoral Collaboration [9, 27, 28]. Each component plays a critical role in promoting the principles of One Health and addressing complex health challenges at the interface of humans, animals, and ecosystems. However, inherent within these elements lie significant challenges that impede their effectiveness and implementation.

##### Stakeholder engagement

Stakeholder engagement is crucial in the One Health framework, fostering collaboration among various actors [8–11]. Government agencies shape policies, healthcare and veterinary professionals provide expertise, NGOs advocate for affected communities, and academic institutions conduct research. International organizations like the WHO, FAO, and OIE coordinate global efforts.

Challenges in stakeholder engagement include jurisdictional conflicts, which hinder collaboration and data sharing [8–11]. Clearing roles, improving communication, and fostering trust are essential to overcome obstacles. Despite supportive policies, implementation faces bureaucratic complexities and competing interests [10, 11]. Legal barriers complicate cross-sectoral cooperation, necessitating aligned frameworks to support integration effectively. Addressing these challenges is vital for realizing the full potential of the One Health approach in addressing complex health issues involving humans, animals, and the environment.

##### Robust institutional frameworks

Within the One Health paradigm, robust institutional frameworks are vital for coordinating policies and interventions across human, animal, and environmental health domains [12, 13]. These frameworks facilitate collaboration among stakeholders, harmonize standards, and enhance intervention efficiency.

However, their effectiveness is hampered by insufficient funding, limiting capacity, and sustainability [12, 13]. Advocacy for increased funding, diversification of sources, and strategic resource allocation are essential for resilience and sustainability. Furthermore, inadequate data sharing and surveillance pose challenges [13]. Incomplete systems impede disease monitoring, while standardized evaluation frameworks are needed to assess intervention impact within these frameworks.

##### Adaptive policies

In One Health governance, adaptive policies are crucial for tackling evolving challenges spanning human, animal, and environmental health domains [8, 33]. These policies are characterized by flexibility and responsiveness, incorporating emerging evidence and adjusting strategies accordingly. Yet, their effectiveness faces obstacles, primarily due to insufficient funding [8, 33].

Limited financial resources hinder essential research, surveillance system establishment, and intervention implementation, compromising disease monitoring and timely response capabilities. Moreover, budgetary constraints curtail adaptive policy initiatives’ scope and innovation potential [8, 33]. Dependency on external funding sources introduces instability and may undermine local autonomy, hindering sustainability efforts. Institutional resistance and bureaucratic inertia further impede adaptive policy adoption, necessitating a culture of innovation and interdisciplinary collaboration.

Overcoming funding challenges, institutional resistance, and fostering collaboration are essential in One Health governance. Mobilizing resources, enhancing capacities, and fostering partnerships are crucial for resilient health systems. Global collaboration is vital for tackling transboundary issues, despite regulatory and political complexities [31].

##### Inter-sectoral collaboration

In the One Health framework, inter-sectoral collaboration is pivotal for cohesive governance, fostering joint efforts across health, veterinary medicine, agriculture, and the environment [9, 27, 28]. This approach recognizes the interconnectedness of health systems and advocates collective action to address complex challenges spanning humans, animals, and ecosystems. However, notable challenges hinder effective collaboration, particularly in cross-disciplinary communication and education [3, 9, 21].

Disparities in language, terminology, and culture hinder communication among stakeholders. Limited interdisciplinary training exacerbates challenges, hindering understanding and collaboration. Efforts to promote training programs, platforms, and mutual respect are crucial.

Addressing capacity and awareness gaps is also critical. Limited understanding of the One Health approach and inadequate training hinder effective implementation. Mobilizing resources and promoting collaboration can enhance the efficacy and sustainability of One Health initiatives, improving health outcomes for all.

### Limitations in the reviewed studies

The systematic exploration of One Health governance mechanisms brought to light several limitations across the scrutinized studies, each impacting the depth and applicability of the findings. Table 2 highlights the limitations and underscores the intricacies of amalgamating diverse research approaches and the inherent challenges in examining multifaceted governance structures.

**Table 2.** Limitations in the reviewed studies.

Limitations	Studies
**1. Heterogeneity in Methodologies:** Each of these studies employs distinct methodologies reflective of their respective research questions and contexts. Such diversity in approach may hinder straightforward comparisons and synthesis of findings. Thus, careful consideration of methodological differences is necessary when interpreting and synthesizing results across studies in the field.	a) Rajan *et al*. [9] b) Simen-Kapeu *et al*. [8] c) Abbas *et al*. [10] d) Aggarwal *et al*. [11]
**2. Limited Geographical Scope:** The focus of these studies is confined to specific regions, namely Thailand and South Asia, respectively. Consequently, the transferability and applicability of their findings on governance mechanism on One Health to a wider global context may be limited. While these studies provide valuable insights into localized contexts and issues surrounding One Health and its governance mechanism, caution should be exercised when extrapolating their conclusions to broader geographical and cultural settings. Thus, acknowledging the geographical limitations of these studies is crucial when interpreting and applying their results in a global context.	a) Rajan *et al*. [9] b) Dahal *et al*. [27]
**3. Contextual Specificity:** These studies focus on specific contexts such as One Health governance within China’s health system and the global health security agenda, respectively. As a result, the transferability of their findings to other settings or environments may be limited. While these studies offer valuable insights into localized issues and approaches to the global health security agenda and what role does governance mechanism in One Health plays, caution should be exercised when extrapolating their conclusions to different geographical, cultural, or institutional contexts. Recognizing the contextual specificity of these studies is essential for appropriately interpreting and applying their findings in diverse settings.	a) Huang *et al*. [12] b) Jenkins [39]
**4. The inclusion of diverse topics within the One Health framework.** These studies cover a wide range of topics within governance mechanisms for the One Health framework, which may result in a shallower analysis of each governance mechanism. While the breadth of topics addressed is valuable for understanding the multifaceted nature of the governance within One Health, it may also limit the depth of examination for individual governance mechanisms. Careful consideration of the scope and focus of each study is necessary to appreciate the nuances and complexities of governance within the One Health paradigm.	a) Huang *et al*. [12] b) Simen-Kapeu *et al*. [8] c) Li *et al*. [14]
**5. The issue of incomplete comprehensive coverage within One Health governance mechanisms:** These studies may not comprehensively cover all aspects or dimensions of One Health governance mechanisms, potentially overlooking crucial elements or strategies. While they contribute valuable insights into specific aspects of governance within the One Health framework, the lack of comprehensive coverage may limit a thorough understanding of the governance mechanisms needed to address complex health challenges at the human-animal-environment interface. Awareness of this limitation is important when interpreting the findings of these studies and when considering strategies for enhancing governance mechanisms within the One Health approach. Efforts to address gaps in coverage and to promote holistic approaches to governance are essential for effective One Health implementation and the mitigation of emerging health threats.	a) Allen [5] b) Huang *et al*. [12]
**6. Timeframe Limitations:** These studies’ limited timeframe may overlook evolving dynamics and recent developments within the One Health governance landscape. To comprehensively assess the effectiveness and sustainability of One Health governance mechanisms, it is crucial to consider studies that encompass a broader and more current timeframe, thus capturing recent trends and emerging issues in the field.	a) Bordier *et al*. [13] b) Kloeze *et al*. [36] c) Li *et al*. [28]
**7. The absence of direct comparative analyses between different governance mechanisms:** The lack of comparative analyses in these studies hinders the ability to assess the relative effectiveness or weaknesses of different governance mechanisms within the One Health framework. Without direct comparisons, it becomes challenging to identify best practices, areas for improvement, and optimal strategies for enhancing One Health governance. In future research endeavours, incorporating comparative analyses between various governance mechanisms would provide valuable insights into their performance and efficacy. Such analyses could inform policymakers, practitioners, and stakeholders in developing more effective and sustainable approaches to One Health governance.	a) Allen [5] b) Huang *et al*. [12] c) Bordier *et al*. [13]

### Implication and importance

The studies highlight the vital role of coordinated governance structures in promoting sustainable health and environmental outcomes within One Health [12, 13]. Huang *et al*. [12] emphasized managing chronic conditions, while Bordier *et al*. [13] stressed cross-disciplinary approaches. Stakeholder engagement is crucial, enhancing resilience and patient-centered care, as noted by Simen-Kapeu *et al*. [8] and Aggarwal *et al*. [11]. Abbas *et al*. [10] and Dahal *et al*. [3] underscored collaborative governance.

These studies underscore integrated governance systems across sectors for effective health management [28]. Effective mechanisms ensure equitable access and cross-sectoral collaboration, fostering community engagement and international cooperation, as emphasized by Li *et al*. [28], Rajan *et al*. [9], and Liu *et al*. [33].

### Limitations of the systematic review and methodology

Methodological Diversity and Varied Approaches: The presence of methodological diversity across reviewed studies hindered direct comparisons and robust assessments [9, 12, 13, 28, 34]. This heterogeneity may limit the generalizability of conclusions drawn.

Challenges in National-level Policy Integration: Persistent hurdles in effectively integrating policies and operationalizing One Health governance strategies at the national level were noted. Overcoming bureaucratic frameworks and aligning diverse stakeholder interests pose substantial obstacles [9].

### Recommendations for future research

- **Standardizing methodologies:** Future research should promote uniform methodologies across studies for consistency and comparability, improving reliability and generalizability of findings [12, 13, 28].
- **Strategies for policy integration:** Research should explore innovative approaches to overcome policy integration hurdles at the national level, enhancing effective governance [9].
- **Interdisciplinary collaboration and capacity building:** Emphasizing interdisciplinary collaboration and capacity-building initiatives across human, animal, and environmental health sectors is essential for effective integrated approaches [33].
- **Evaluation frameworks:** Developing evaluation frameworks for One Health interventions is crucial. Standardized frameworks would enable comprehensive assessments [12].
- **Cross-sectoral dialogue and collaboration:** Promoting dialogue and collaboration between countries and stakeholders should focus on aligning legal frameworks for smoother cooperation [13].

## Conclusion

This systematic review comprehensively explored governance architectures within the domain of One Health, shedding light on their effectiveness, sustainability, stakeholder engagement, and potential challenges. Robust governance mechanisms emerged as fundamental in orchestrating collaborative efforts essential for the success and sustainability of One Health initiatives. Stakeholder engagement, integrated governance systems, policy frameworks, and cross-sectoral collaboration were identified as crucial elements in addressing complex health challenges.

Addressing the highlighted challenges and limitations requires concerted efforts from governments, international organizations, and stakeholders. Despite valuable insights offered by these studies, certain limitations were noted, including methodological diversity and challenges in policy integration. Future research endeavours should focus on standardizing methodologies, exploring strategies for policy integration, emphasizing interdisciplinary collaboration, developing evaluation frameworks, and promoting cross-sectoral dialogue and collaboration to enhance the effectiveness and sustainability of integrated health approaches within the One Health framework.

## Reflexivity statement

The reviewer team is experienced in diverse research skills, including both primary research in qualitative and quantitative methods, and evidence synthesis methods for quantitative study designs. They have a rich working as well as research experience in fields of infectious diseases (D.D.F., O.D., L.M., K.S., A.R.), clinical medicine (K.S., A.R.), clinical trials (D.D.F.), TB and HIV management (D.D.F., K.S.), social science (K.S., A.O.S.), and One Health (O.D., L.M., K.S.). The team reflected on personal biases or judgments throughout the process of the synthesis. This allowed them to reflect on emerging themes through different perspectives and to construct meaning in the synthesis. To further increase reflexivity, “negative” cases, that is, findings that conflicted or offered alternate explanations to emerging themes or to the conceptual model, were carefully considered.

## Supplementary Material

ckae124_Supplementary_Data

## Data Availability

The data used to support the findings of this study can be made available upon reasonable request. Key points The systematic review aimed to evaluate governance architectures within the One Health domain, nine selected studies were analysed focusing on effectiveness, sustainability, stakeholder engagement, and challenges. Stakeholder engagement, integrated governance systems, policy frameworks, and cross-sectoral collaboration emerged as crucial elements for addressing complex health challenges. The review offers valuable insights for policymakers, practitioners, and researchers, highlighting the need for integrated approaches to global health management. Limitations include methodological heterogeneity, limited geographical scope, and reliance on qualitative or review approaches in some studies. Challenges in integrating policies and implementing One Health at the national level include intersectoral collaboration, funding constraints, fragmented governance structures, inadequate data sharing, capacity gaps, and conflicts among stakeholders.
