# Supplementary material for: Constructing a One Health governance architecture: a systematic review and analysis of governance mechanisms for One Health
Source: Eur J Public Health. 2024 Aug 30;34(6):1086–94. doi: 10.1093/eurpub/ckae124 (PMC11631453; doi:10.1093/eurpub/ckae124)
Supplement: ckae124_Supplementary_Data [file ckae124_supplementary_data.zip › ckae124_Supplementary_Data/ejph-2024-05-om-0295-File004.pdf]

Supplementary Table I: MeSH Terms and Keywords Related to Governance and Policy in One Health and Interdisciplinary Health Approaches.

No.	MeSH Terms:	Combined Number of Articles Yielded from PubMed, Scopus, Web of Science (WoS), and Cochrane
1.	"One Health governance,"	150
2.	“Interdisciplinary health governance,"	100
3.	“Governance mechanisms for One Health,"	121
4.	"Integrated health governance,"	79
5.	"Policy frameworks for One Health,"	146
6.	"Collaborative strategies in One Health,"	104
7.	"Stakeholder engagement in One Health,"	63
8.	"Environmental health governance,"	137
9.	“Human-animal health governance,"	112
10.	"Public health policy and One Health," and	90
11.	"Global health governance strategies."	87

Supplementary Table II. Themes and Key Findings from Reviewed Studies

**Theme**	**Key Findings**	**Example Studies**
Engagement Mechanism	Emphasized stakeholder engagement in health decision-making and community participation	Aggarwal et al. (2022) [12]<br>Rajan et al. (2019) [28]
Coordination Mechanism	Highlighted the impact of coordinated governance structures on health outcomes	Dahal et al. (2017) [30]<br>Huang et al. (2016) [9]<br>Abbas et al., 2022) [11]
Policies Mechanism	Discussed the efficacy of health policies in improving governance and health outcomes	Simen-Kapeu et al. (2021) [15]<br>Liu et al. (2023) [16]<br>Boucher et al. (2016) [29]
Financial Mechanism	Explored financial strategies to support One Health initiatives	Huang et al. (2016) [13]<br>Bordier et al. (2020) [14]

Supplementary Table III. Overview of Reviewed Studies

First Author (Reference)	Region	Focus	Methodology	Key findings
Huang et al. (2016) [13]	China	Coordinated governance	Qualitative analysis	Coordinated governance structures were pivotal, highlighting the necessity of collaborative frameworks for sustainable health outcomes, especially in managing chronic health conditions.
Dahal et al. (2017) [30]	South Asia	Cross-sectoral collaboration	Literature reviews and stakeholder consultations.	This study underscored the significance of cross-sectoral collaboration and stakeholder engagement within governance frameworks for effective implementation of One Health approaches
Rajan et al. (2019) [28]	Thailand	Community engagement	Participatory action research and stakeholder workshops.	This study highlighted the role of participatory governance structures in ensuring community engagement and inclusivity in health decision-making processes.
Bordier et al. (2020) [14]	Globally	Collaborative strategies	Systematic review analyzing One Health surveillance systems globally, focusing on cross-disciplinary literature analysis.	Emphasized the significance of effective cross-disciplinary approaches and collaborative strategies within governance frameworks for successful surveillance systems and emphasized the need for integrated approaches and stakeholder collaboration.
Simen-Kapeu et al. (2021) [15]	Liberia	Stakeholder consultations.	Qualitative surveys and stakeholder consultations.	Stressed stakeholder engagement within governance structures to enhance health system resilience, particularly in resource-constrained settings. This study also highlights engagement and collaboration in enhancing health system resilience, particularly in resource-constrained settings, emphasizing the importance of participatory approaches within governance frameworks.
Li et al. (2021) [31]	China	Integrated governance systems	Field observations and case studies.	Investigated wild animal and zoonotic disease risk management in China. The study highlighted the imperative of integrated governance systems involving multiple sectors and stakeholders for effective zoonotic disease management.
Abbas et al., 2022) [11]	Globally	Collaborative governance structures	Qualitative interviews and case studies.	The study emphasized the necessity of collaborative governance structures and stakeholder engagement as fundamental components for successful One Health initiatives.
Aggarwal et al. (2022) [12]	Globally	Stakeholder engagement	Mixed-methods surveys and interviews.	Emphasized stakeholder engagement and collaboration within governance structures for patient-centered care and effective health system governance. Explored the role of patients in governing sustainable healthcare systems.
Liu et al. (2023) [16]	BRICS Countries	Governance frameworks and policies	Policy analysis and expert consultations.	The research underscores the pivotal role of governance frameworks and policies in fostering health collaboration across nations, emphasizing

				the significance of collaborative governance mechanisms within the multifaceted realm of One Health. It accentuates the imperative for comprehensive approaches encompassing human, animal, environmental health, and other pertinent dimensions to solve health issues such as zoonotic diseases outbreak, thereby advocating for integrated governance structures that address the interconnectedness of health domains within the One Health paradigm.
