# Supplementary material for: Constructing a One Health governance architecture: a systematic review and analysis of governance mechanisms for One Health
Source: Eur J Public Health. 2024 Aug 30;34(6):1086–94. doi: 10.1093/eurpub/ckae124 (PMC11631453; doi:10.1093/eurpub/ckae124)
Supplement: ckae124_Supplementary_Data [file ckae124_supplementary_data.zip › ckae124_Supplementary_Data/ejph-2024-05-om-0295-File006.pdf]

[41]. Kloeze H, Mukhi S, Kitching P, Lees VW, Alexandersen S. Effective animal health disease surveillance using a network-enabled approach. Transboundary and emerging diseases 2010; 57(6): 414-9.

[42]. Elnaiem, A., Mohamed-Ahmed, O., Zumla, A., Mecaskey, J., Charron, N., Abakar, M. F., Raji, T., Bahalim, A., Manikam, L., Risk, O., Okereke, E., Squires, N., Nkengasong, J., Rüegg, S. R., Abdel Hamid, M. M., Osman, A. Y., Kapata, N., Alders, R., Heymann, D. L., Kock, R., … Dar, O. (2023). Global and regional governance of One Health and implications for global health security. Lancet (London, England), 401(10377), 688–704. https://doi.org/10.1016/S0140-6736(22)01597-

[43]. Sinclair, J. R. (2019). Importance of a One Health approach in advancing global health security and the Sustainable Development Goals. Revue scientifique et technique (International Office of Epizootics), 38(1), 145–154. https://doi.org/10.20506/rst.38.1.2949

[44]. Mazet, J. A., Uhart, M. M., & Keyyu, J. D. (2014). Stakeholders in One Health. Revue scientifique et technique (International Office of Epizootics), 33(2), 443–452. https://doi.org/10.20506/rst.33.2.2295

[45]. Aslam, B., Khurshid, M., Arshad, M. I., Muzammil, S., Rasool, M., Yasmeen, N., Shah, T., Chaudhry, T. H., Rasool, M. H., Shahid, A., Xueshan, X., & Baloch, Z. (2021). Antibiotic Resistance: One Health One World Outlook. Frontiers in cellular and infection microbiology, 11, 771510. https://doi.org/10.3389/fcimb.2021.771510

[46]. Horefti, E. (2023). The Importance of the One Health Concept in Combating Zoonoses. Pathogens (Basel, Switzerland), 12(8), 977. https://doi.org/10.3390/pathogens12080977

[47]. Keusch, G. T., Amuasi, J. H., Anderson, D. E., Daszak, P., Eckerle, I., Field, H., Koopmans, M., Lam, S. K., Das Neves, C. G., Peiris, M., Perlman, S., Wacharapluesadee, S., Yadana, S., & Saif, L. (2022). Pandemic origins and a One Health approach to preparedness and prevention: Solutions based on SARS-CoV-2 and other RNA viruses. Proceedings of the National Academy of Sciences, 119(42). https://doi.org/10.1073/pnas.2202871119

[48]. Espeschit, I. F., Santana, C. M., & Moreira, M. A. S. (2021). Public Policies and One Health in Brazil: The Challenge of the Disarticulation. Frontiers in public health, 9, 644748. https://doi.org/10.3389/fpubh.2021.644748
