# Supplementary figures and images for: Constructing a One Health governance architecture: a systematic review and analysis of governance mechanisms for One Health
Source: Eur J Public Health. 2024 Aug 30;34(6):1086–94. doi: 10.1093/eurpub/ckae124 (PMC11631453; doi:10.1093/eurpub/ckae124)

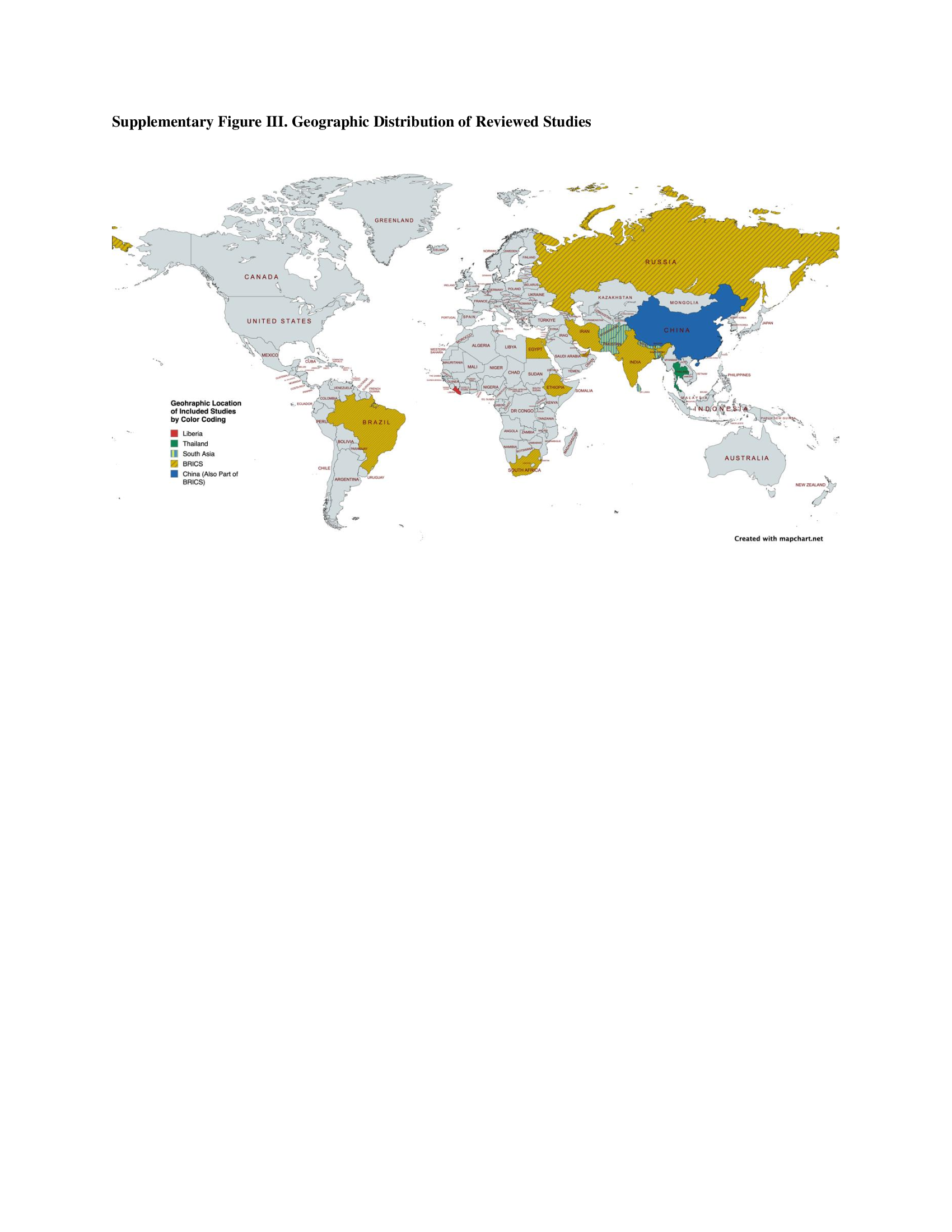

Supplement: ckae124_Supplementary_Data [file ckae124_supplementary_data.zip › ckae124_Supplementary_Data/ejph-2024-05-om-0295-File005.tiff]
